# Treatment of Extraosseous Giant Cell Tumor of Bone and Calcitriol-Mediated Hypercalcemia With Denosumab in Paget Disease

**DOI:** 10.1210/jcemcr/luaf031

**Published:** 2025-03-19

**Authors:** Oyunbileg Magvanjav, Clemens Bergwitz

**Affiliations:** Section of Endocrinology and Metabolism, Yale University School of Medicine, New Haven, CT 06519, USA; Section of Endocrinology and Metabolism, Yale University School of Medicine, New Haven, CT 06519, USA

**Keywords:** hypercalcemia, Paget disease of bone, giant cell tumor, denosumab, calcitriol, 1,25-dihydroxyvitamin D

## Abstract

Extraosseous giant cell tumor of bone (GCTB) associated with Paget disease of bone (PDB) is rare. We report a patient aged in their 70s with polyostotic PDB involving the skull, spine, and pelvis, previously treated with bisphosphonates, who presented with symptomatic hypercalcemia (calcium 14.8 mg/dL [3.7 mmol/L]; reference range [RR], 8.6-10.5 mg/dL [2.1-2.6 mmol/L]), kidney injury (creatinine 2.6 mg/dL [230 μmol/L]; RR, 0.4-1.1 mg/dL [35-97 μmol/L]), and a 17.5 cm pelvic mass. Testing showed elevated calcitriol or 1,25-dihydroxyvitamin D (1,25(OH)_2_D) (57-108 pg/mL [137-259 pmol/L]; RR, 18-72 pg/mL [43-173 pmol/L]), but normal parathyroid hormone and bone-specific alkaline phosphatase (BSAP), arguing against parathyroid autonomy and active osseous PDB. Histopathology showed osteoclast-like giant cells and stromal mononuclear cells without atypia, necrosis, or mitoses. A one-time dose of denosumab 120 mg resulted in normalized calcium (9.0 mg/dL [2.2 mmol/L]) and 1,25(OH)_2_D (24 pg/mL [57 pmol/L]) and reduced tumor size. Denosumab was continued at a dose of 60 mg every 6 months. After 20 months, calcium and 1,25(OH)_2_D remained normal, with no tumor regrowth, and BSAP stayed low. This is the first report of 1,25(OH)_2_D-mediated hypercalcemia in extraosseous GCTB. It responded well to denosumab. Long-term management options are discussed in the context of existing literature.

## Introduction

Giant cell tumor of bone (GCTB) is a rare, locally aggressive osteolytic neoplasm that often occurs in the pelvis or axial skeleton [1, 2]. Malignant forms account for < 10% of GCTBs [3, 4]. GCTB can occur in < 1% of patients with Paget disease of bone (PDB), a disorder of disorganized bone remodeling [1, 5, 6]. GCTB in PDB primarily occurs in bones affected by PDB but may rarely arise from tissues adjacent to the PDB-affected bone. In a review of PDB cases complicated by GCTB (n = 117), 94% of GCTB occurred in the bone, while 6% occurred at extraosseous sites (buttock, knee joints, skull soft tissue, sinuses) [1]. Several genes are implicated in GCTB (*H3F3A, CSF1, ZNF687, PFN1*) and PDB (*SQSTM1, TNFRSF11A, ZNF687, TNFRSF11B, DCSTAMP, OPTN, CSF1, VCP, HLA, FKBP5, NUP205, PML, RIN3*); the most common variants are *H3F3A*.p.G34W in GCTB and *SQSTM1*.p.P392L in PDB [7, 8]. The *ZNF687*.p.P937R pathogenic variant has been linked to both GCTB and PDB [9]. GCTB is characterized by osteoblast-like stromal cells that express RANKL, attracting osteoclast-like giant cells that cause osteolysis [1, 6]. Osteoclasts also express CYP27B1, which may promote bone resorption [10].

GCTB and PDB rarely cause hypercalcemia unless other conditions, such as hyperparathyroidism, are present. PDB is typically treated with bisphosphonates, while surgery is first-line for GCTB, with antiresorptive agents, radiation, or embolization for inoperable/refractory cases [2, 11-14]. Clinical trials suggest denosumab at a dose of 120 mg monthly in nonoperable GCTB [11, 15, 16]. Here, we present a patient with multifocal PDB, 1,25-dihydroxyvitamin D (1,25(OH)_2_D)-induced hypercalcemia, and a large, inoperable extraosseous GCTB, in whom denosumab was highly effective in inducing remission of both the hypercalcemia and GCTB. We discuss long-term management options considering the current literature.

## Case Presentation

A patient in their 70s presented with fatigue, abdominal discomfort, weakness, and polyuria of several weeks. Medical history included PDB, asthma, hypertension, chronic kidney disease (CKD)-III, anemia, gout, and remote right ankle fracture. Medications included amlodipine, epoetin, albuterol, montelukast, and fluticasone-vilanterol, and no supplemental vitamin D/calcium. The patient was diagnosed with PDB in their 50s and treated with bisphosphonates (alendronate for 5 years until 10 years prior; zoledronate for 3 years until 3 years prior). The patient is of European-Italian descent and has a daughter who has achondroplasia. The parents are unrelated, and there is no family history of PDB/GCTB. Examination revealed normal vital signs and a firm, nontender mass in the left lower abdominal quadrant.

## Diagnostic Assessment

**Figure 1.**
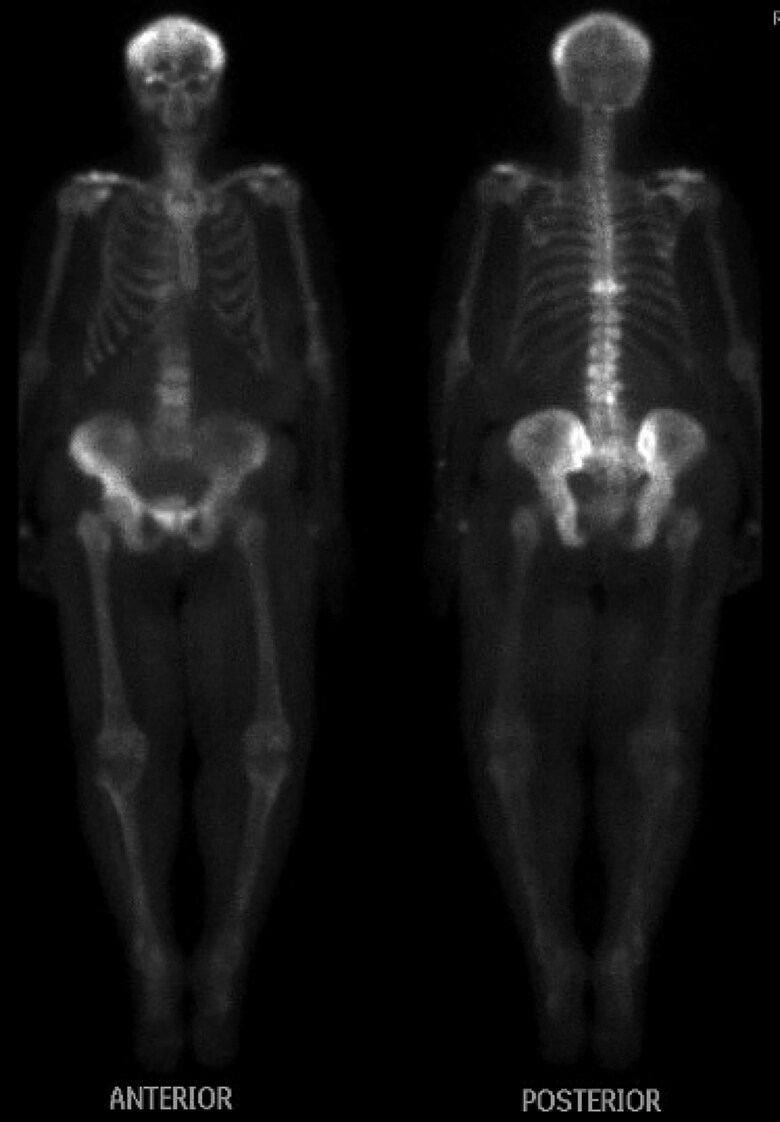
Nuclear medicine whole-body bone scan showing diffuse radiotracer uptake (21 mCi technetium-99 m methylene diphosphonate) in the skull, thoracolumbar spine, and bilateral pelvis. Uptake in the knees and shoulders is consistent with degenerative changes.

**Figure 2.**
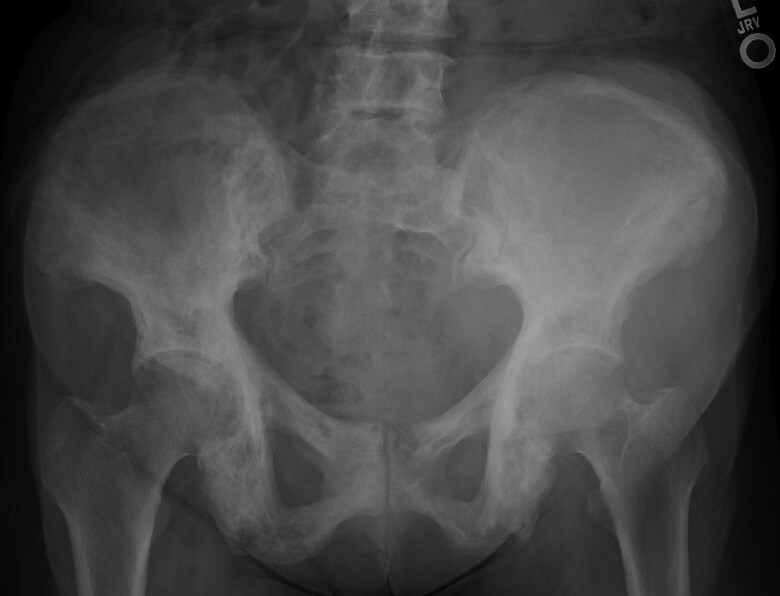
Pelvic x-ray with coarse trabecula and cortical thickening in bilateral pelvic bones suggest Paget disease.

**Figure 3.**
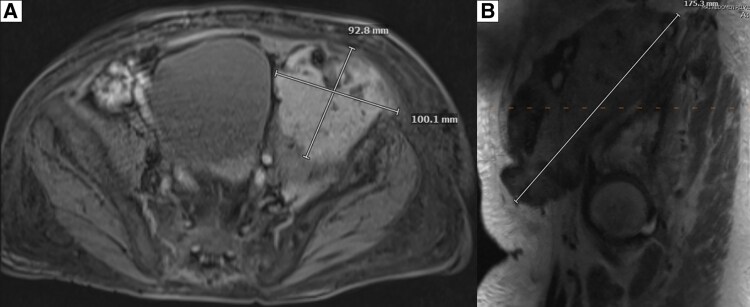
On the initial presentation, the magnetic resonance imaging (MRI) of the abdomen/pelvis shows a large left-sided pelvic mass measuring 10.1 cm × 9.3 cm transaxial (A) and 17.6 cm craniocaudal (B); dimensions are per formal radiology report.

**Figure 4.**
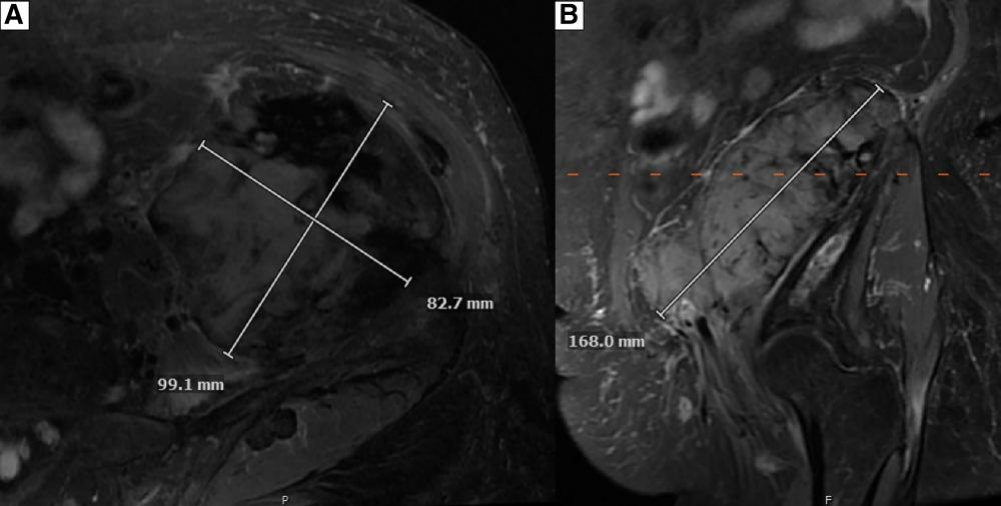
Magnetic resonance imaging (MRI) of the pelvic mass at 8 months before hospitalization with hypercalcemia. (A) transaxial (B) craniocaudal.

Baseline testing showed hypercalcemia (calcium 14.8 mg/dL; 3.7 mmol/L) (reference range [RR], 8.6-10.5 mg/dL; 2.1-2.6 mmol/L), acute kidney injury (AKI) (creatinine 2.6 mg/dL; 230 μmol/L) on CKD (baseline creatinine ∼1.7 mg/dL; ∼150 μmol/L) (RR, 0.4-1.1 mg/dL; 35-97 μmol/L), and elevated 1,25(OH)_2_D (108 pg/mL; 259 pmol/L) (RR, 18-72 pg/mL; 43-173 pmol/L) (Table 1). Workup excluded sarcoidosis, hyperthyroidism, and multiple myeloma, with normal angiotensin-converting enzyme, thyroid stimulating hormone, and protein electrophoresis, respectively. A malignancy workup was negative for carcinoembryonic antigen (CEA), CA-19-9, and CA-125. Imaging showed no osteolytic bone metastasis, with tracer uptake consistent with PDB in the skull, thoracolumbar spine, and pelvis on a whole-body bone scan (Fig. 1). Pelvic x-ray showed PDB in the lumbar/pelvic regions; no x-rays were available of the skull or entire spine (Fig. 2). Magnetic resonance imaging (MRI) revealed a 10.1 × 9.3 × 17.6 cm intrapelvic retroperitoneal mass along the left iliopsoas muscle, abutting the left iliac crest (but not involving the iliac bone), extending into the left groin, medially displacing the left external iliac vasculature, and abutting the left iliac artery (Fig. 3). The mass was stable in comparison with imaging obtained 8 months earlier (Fig. 4) (10.1 × 8.1 cm [transaxial] × 13.5 cm [cranio-caudal]); comparative re-reads provided estimates closer to 16 to 17 cm cranio-caudally, indicating that the mass likely had always been ∼17 cm cranio-caudally.

**Table 1.** Patient’s initial laboratory test results on admission

Test	Value	Reference range
Serum calcium	**14.8 mg/dL** (3.7 mmol/L)	8.6-10.5 mg/dL (2.1-2.6 mmol/L)
Serum albumin	4.2 g/dL (0.6 mmol/L)	3.5-5.2 g/dL (0.5-0.8 mmol/L)
Serum phosphorus	3.4 mg/dL (1.1 mmol/L)	2.2-4.5 mg/dL (0.7-1.4 mmol/L)
Serum creatinine	**2.0-2.6 mg/dL** *^a^* (177.0-230.0 μmol/L)	0.4-1.1 mg/dL (35.0-97.0 μmol/L)
PTH	19.0 pg/mL (2.0 pmol/L)	15.0-65.0 pg/mL (1.6-6.9 pmol/L)
PTHrP	12.0 pg/mL (1.3 pmol/L)	11.0-20.0 pg/mL (1.2-2.1 pmol/L)
25(OH)D	35.0 ng/mL (87.0 nmol/L)	20.0-50.0 ng/mL (50.0-125.0 nmol/L)
1,25(OH)_2_D	**57.0-108.0 pg/mL** *^b^* (137.0-259.0 pmol/mL)	18.0-72.0 pg/mL (43.0-173.0 pmol/L)
Bone-specific ALP	10.5 μg/L	5.6-29.0 μg/L

Abnormal values are in bold font. Values in International System of Units (SI) are provided in parentheses where needed.

Abbreviations: 1,25(OH)2D, 1,25-dihydroxyvitamin D; 25(OH)D, 25-hydroxyvitamin D; AKI, acute kidney injury; ALP, alkaline phosphatase; CKD, chronic kidney disease; PTH, parathyroid hormone; PTHrP, parathyroid hormone-related peptide.

^a^The patient’s serum creatinine on admission demonstrated AKI on CKD, ranging from 2.0-2.6 mg/dL (177.0-230.0 μmol/L) during the hospitalization, which was above their baseline of ∼1.7 mg/dL (150.0 μmol/L).

^b^The baseline 1,25(OH)2D level on admission was elevated to 108.0 pg/mL (259.0 pmol/L), which later decreased to a high normal level of 57.0-65.0 pg/mL (137.0-156.0 pmol/L) during their hospitalization.

**Figure 5.**
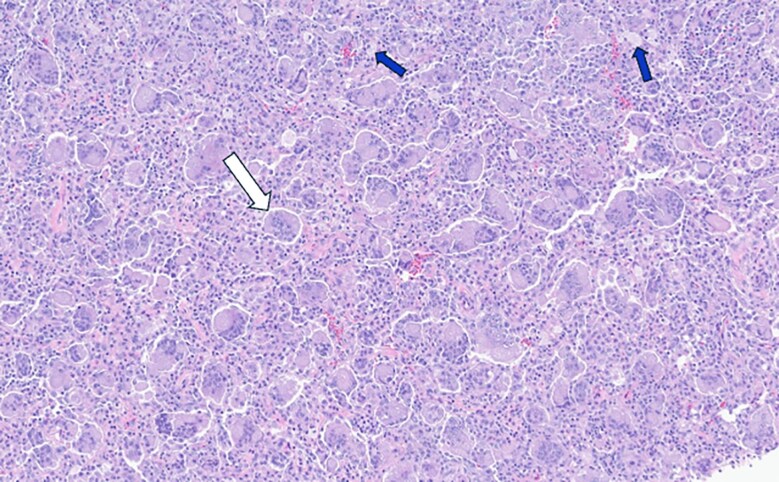
Hematoxylin and eosin staining of pelvic mass biopsy specimen shows uniformly distributed multi-nucleated osteoclast-like giant cells (example indicated by the white arrow) on a background of homogenous mononuclear stromal cells and foamy histiocytes (blue arrows); no appreciable mitotic activity, significant cytologic atypia nor necrosis is identified.

The patient first discovered the pelvic mass upon self-palpation 1 year before presenting and was otherwise asymptomatic. Testing showed a slightly elevated calcium (11.2 mg/dL; 2.8 mmol/L) with concomitant AKI (creatinine 2.5 mg/dL; 224 μmol/L), suppressed parathyroid hormone PTH (8.8 pg/mL; 0.9 pmol/L) (RR, 18-88 pg/mL; 1.9-9.3 pmol/L), and normal 25(OH)D (58.8 ng/mL; 147 nmol/L) (RR, 30-100 mg/mL; 75-250 nmol/L). Tumor biopsy revealed a benign GCTB (Fig. 5). Tumor genetics revealed a likely benign missense variant (*CBL*.pC404Y) and was negative for pathogenic variants in *S100, SOX10, H3, MDM2, CDK4, BRAF VE1,* and *CD1a*. Histology showed negative pancytokeratin staining; Ki67 index 10% to 15%. Imaging indicated that the tumor was not growing. As the patient was asymptomatic, surgery was held off. Calcium was normal (10.1 mg/dL; 2.5 mmol/L) on the latest check 3 months prior to presenting with symptomatic hypercalcemia.

## Treatment

Over one week in the hospital, the patient received 6 L of intravenous fluids (100-150 mL/hour), nasal calcitonin 200 units daily (3 doses), and pamidronate 30 mg daily (2 doses). Calcium decreased from 14.8 mg/dL (3.7 mmol/L) to 11.0 mg/dL (2.7 mmol/L). On day 13, prednisone 40 mg daily was started due to the elevated 1,25(OH)_2_D on admission. After 2 days, calcium only slightly decreased from 11.4 mg/dL (2.8 mmol/L) to 10.7 mg/dL (2.7 mmol/L). Denosumab 120 mg was then given, and calcium normalized to 10.1 mg/dL (2.5 mmol/L), reaching 9.0 mg/dL (2.2 mmol/L) on day 18. The cause of hypercalcemia and elevated 1,25(OH)_2_D remained unclear but was suspected to be related to the tumor. The case was discussed at multidisciplinary tumor board, and due to the tumor's size, location, and surrounding tissue involvement, surgery was deferred, and conservative management was recommended.

## Outcome and Follow-Up

**Figure 6.**
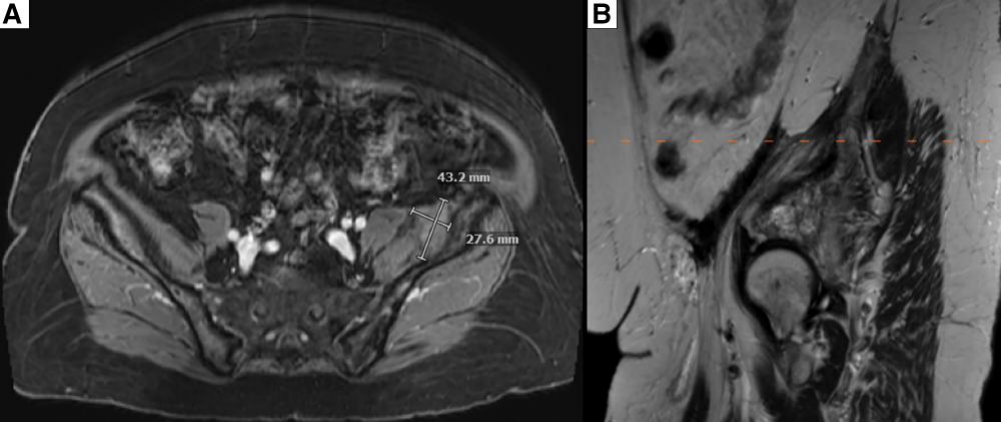
Pelvic mass after 20 months of denosumab therapy^1^ shows a substantial decrease in size of the GCTB to 4.3 × 2.8 cm. Abbreviation: GCTB, giant cell tumor of bone. ^1^Denosumab therapy consisted of 120 mg (first dose) followed by 60 mg every 6 months.

Eight months after the first denosumab dose, the tumor had shrunk to 5.7 × 3.7 cm. After 20 months, it further shrank to 4.3 × 2.8 cm (Fig. 6). The patient has continued to receive denosumab 60 mg every 6 months for 2 years. Calcium remains normal, creatinine stable (∼1.9 mg/dL; ∼168 μmol/L), and 1,25(OH)_2_D, collagen-c-telopeptide (CTx), and bone-specific alkaline phosphatase (BSAP) are low/normal at 19 pg/mL (46 pmol/L), 126 pg/L (no RR established), and 8.6 µg/L (RR, 5.6-29 µg/L; SI units are the same), respectively. Disease remains controlled per laboratory testing and imaging.

## Discussion

We present a rare case of a patient with GCTB-PDB with hypercalcemia from elevated 1,25(OH)_2_D. Hypercalcemia occurring in GCTB and/or PDB is atypical. After reviewing the English biomedical literature in PubMed using the search terms *hypercalcemia*, *giant cell*, and *1,25(OH)_2_D*, *1,25-dihydroxyvitamin D*, or *calcitriol*, only 9 articles returned; none described 1,25(OH)_2_D-mediated hypercalcemia in GCTB or PDB.

Our patient's hypercalcemia was likely from elevated 1,25(OH)_2_D, increasing calcium by enhancing intestinal absorption. Bone turnover from Pagetic lesions or direct osteoclastogenic activity from 1,25(OH)_2_D are less likely causes—these would typically elevate BSAP. Cytokine-mediated bone resorption is another consideration [17, 18]; we do not have a pretreatment CTx level. Extraosseous tumors may more likely cause hypercalcemia through humoral mechanisms, such as 1,25(OH)_2_D production or cytokine-mediated bone resorption [19]. In our case, the source of the elevated 1,25(OH)_2_D was likely the GCTB, as tumor remission coincided with the resolution of hypervitaminosis D and hypercalcemia, and other potential sources, such as sarcoidosis or lymphoma, were excluded. Soft tissue sarcomas have been reported to cause paraneoplastic 1,25(OH)_2_D-mediated hypercalcemia [20]. Additionally, renal impairment may have reduced calcium clearance, as seen in Milk-alkali syndrome [21]. The AKI on CKD may have further contributed to hypercalcemia.

Surgery is first-line treatment for GCTB, but recurrence is high, particularly with curettage (>50%) than en bloc resection (0%-12%) [22, 23]. For unresectable tumors, denosumab and, to a lesser extent, zoledronic acid have been used. A study of 54 patients with unresectable GCTB treated with denosumab showed tumor control and improved functionality in all. Of those who discontinued therapy, 40% experienced tumor progression after a median of 8 months [24]. In a follow-up study of 532 patients, among the 262 unresectable cases, 11% had tumor recurrence after a median follow-up of 58 months [11]. These findings and others led to the Food and Drug Administration approval of denosumab for nonresectable or metastatic GCTB [25]. In trials on denosumab for GCTB, 120 mg was given monthly until disease recurrence [15, 16]. In our study, we were able to substantially shrink the patient's tumor using 120 mg (initial dose), followed by 60 mg given every 6 months, which maintained remission. Lastly, optimal treatment duration remains unclear. Long-term use carries risks, including osteonecrosis of the jaw (3%) and atypical femoral fractures (1%) [11].

Bisphosphonates have also shown efficacy, though more extensive trials for nonresectable tumors are limited. Smaller studies suggest bisphosphonates reduce recurrence when administered perioperatively. Among 44 patients undergoing surgery, recurrence was 4% (1/24) in the bisphosphonate-treated group vs 30% (6/20) among controls [26]. Another study evaluating preoperative zoledronic acid showed 5% (1/18) recurrence in the zoledronic acid group vs 21% (4/19) among controls; difference was statistically nonsignificant [27].

In terms of long-term maintenance therapy, the optimal regimen to keep GCTB in remission remains unclear, with few studies directly comparing denosumab and zoledronic acid. Among 160 patients with nonresectable GCTB randomized to receive denosumab (120 mg every 4 weeks) or zoledronic acid (4 mg every 4 weeks), there was no significant difference in tumor response between groups, but cumulative recurrence-free survival was higher in the denosumab group at the end of 3 years [28]. Sequential therapy may be another option, with one case study showing successful remission with denosumab for induction followed by zoledronic acid for maintenance, with no tumor recurrence while off therapy after 10 months of follow-up [29]. To our knowledge, bisphosphonate and denosumab have not been given simultaneously, which may be a third option to stabilize our patient's calcium level and tumor size.

We present a patient in their 70s with multifocal PDB, hypercalcemia, and a large, nonresectable GCTB with elevated 1,25(OH)2D. Hypercalcemia resolved with denosumab, which also significantly reduced the tumor size. Two years later, the tumor remains stable after denosumab 120 mg (initial dose), followed by 60 mg every 6 months. Denosumab is standard of care for inoperable GCTB. Maintenance therapy could include long-term denosumab alone or followed by zoledronic acid with a drug holiday. Comparative studies of maintenance strategies are needed. Tumor regrowth should prompt consideration of malignant transformation. Surgery should be considered first but re-dosing with denosumab is a viable option. Our case adds to the growing evidence supporting denosumab as a well-tolerated option for inoperable GCTB.

## Learning Points

- Extraosseous GCTB in PDB co-occurring with 1,25(OH)_2_D-mediated hypercalcemia has not been reported.
- Denosumab is a feasible well-tolerated treatment for both 1,25(OH)2D-mediated hypercalcemia and GCTB.
- Further studies are needed comparing the efficacy and safety of denosumab vs bisphosphonate, alone or sequentially, as long-term maintenance therapy for nonresectable GCTB.

## Data Availability

Data sharing does not apply to this article as no datasets were generated or analyzed during the current study.

## Abbreviations

1,25(OH)_2_D: 1,25-dihydroxyvitamin D
AKI: acute kidney injury
BSAP: bone-specific alkaline phosphatase
CKD: chronic kidney disease
CTx: collagen-c-telopeptide
GCTB: giant cell tumor of bone
MRI: magnetic resonance imaging
PDB: Paget disease of bone
RR: reference range
